# Diathesis and Cachexiæ during Intercurrent Attacks—The Importance of Their Recognition and Treatment

**Published:** 1890-09

**Authors:** Geo. H. Winston

**Affiliations:** West Point, Ga.


### Southern Medical Record.

A MONTHLY JOURNAL OF MEDICINE AND SURGERY.

VOL. XX. ATLANTA, GA., SEPTEMBER, 1890. NO. 9.

### Original Articles.

### DIATHESIS AND CACHEXIÆ DURING INTERCURRENT ATTACKS—THE IMPORTANCE OF THEIR RECOGNITION AND TREATMENT.

BY GEO. H. WINSTON, M. D., WEST POINT, GA.

It is not the purpose of this paper to discuss the ætiology, cause or treatment of diathesis or cachexiæ, but merely to point out their effects upon intercurrent diseases and the necessity of, where possible, removing or lessening their influences. By an intercurrent disease is here meant any affection that may fasten itself upon an organism already in one of those abnormal states known as a diathesis or cachexiæ; which terms may, for the purposes of this article, be regarded as entirely synonomous. The attack in question may be wholly, in part, or not at all the result of the diathesis.

The tendency of the present day physician is toward seeking for a clear cut diagnosis of some definite disease. The present advanced state of medical science demands that he shall view the symptoms, determine a cause, and treat accordingly. In his endeavor to do so he is too often liable to overlook certain depraved conditions which may exist in the individual, and which will have a greater or less effect upon the course and termination of the process that he has undertaken to treat. He sees a disease, diagnoses and successfully treats it. In a short time views the same symptoms, gives the same treatment, and ignominiously fails to produce any good effect. Why? Different temperaments in different patients, different degrees of virulence in the morbid action, different surroundings all contribute toward the explanation; but in many cases the chief cause of the failure lies in some diathesis that, if not wholly, or in part, the direct cause of the attack, weakens nature and renders her unfit to cope with it. The presence of these evils is often not recognized; not so much from ignorance, for every competent physician is conversant with their symptoms, as from carelessness due to a non-appreciation of their gravity. More often they are recognized and ignored. The whole attention is directed to the apparent urgent symptoms of the acute attack and the physician goes to patching the roof while entirely neglecting the rotten foundations. Too often it lies with providence whether or not the whole shall fall beyond the possibility of repair.

As before stated, every practicing physician should be, and probably is, able to recognize the existence of a diathesis or cachexia. It is the author's chief purpose to impress the important fact, that these conditions, wherever met, should always if possible, be treated. Hence but a few of the more common of these conditions will be considered specially, and they will be barely touched upon their leading points of diagnosis and treatment. In this region fully nine-tenths of these conditions will be included in the following list. I. The syphilitic. II. The rachitic. III. The strumous. IV. The gouty. V. The malarial. VI. The scorbutic.

The man who has acquired or inherited a syphilitic diathesis leads a precarious existence. Apart from the diseases of the nervous and osseous systems to which he is so peculiarly liable, his system seems especially liable to fall before any acute attack. His organs are in a state of, if I may use the term, sub-cinhosis. He lives upon a lowered plane of vitality, and disease either annihilates him at the first blow, or clings to his system day after day with a pertinacity that seems to defy every effort to dislodge it. And yet, if the diathesis be recognized and treated in time, these intercurrent affections, when in themselves amenable to treatment, will, as a rule rapidly disappear before appropriate remedies. I place this diathesis at the head of my list because it appears to me to be by far the most common. Either acquired or inherited, it is largely distributed among the whites, while among the colored population its prevalence is enormous. Among the latter it is, as a rule, either badly treated or not treated at all, and it is among them that its effects are most disastrous. The diagnosis is usually easy. The skin will show evidence of old syphiloderma. Glandular enlargement, more or less morbid, is always present. The post-cervical glands are usually to be felt. I am accustomed to place most reliance upon the evidence of an enlarged epitroclear gland; if I find this gland enlarged, and can trace it to no local cause, be it in infant or in adult, I feel assured of the existence of a syphilitic diathesis, and treat accordingly. When enlarged upon both sides it gives double assurance. It is claimed that syphilis is a self limited disease; but my experience induces me to believe that it leaves when imperfectly treated, a certain condition or diathesis that exists indefinitely, and which will be improved by the use of anti-syphilitic remedies, notably the iodide of potassium. Last August I treated a series of cases of continued fever, the majority of whom ran a regular course of about four weeks. Among them was a man who presented evidences of syphilis, and who gave a history of having had the disease some eighteen years before. His fever continued without amelioration until the thirtieth day, assuming more and more of a typhoid type, when I added potassium iodide, 15 grains, and hydrarg bichloride 1-24 grain given *ter in die*, to my treatment. His improvement was almost immediate, and by the ninth day he was convalescent. Many of our chronic lung affections and many of those acute inflammations that show a tendency to assume a typhoid type have a basis of syphilis. In infants chronic bowel affections are often largely influenced by its presence. On May 24th, 1890, I was called to see a negro child aged one and one-half years. Found long continued diarrhœa; had received various treatments without success; child terribly emaciated, epitroclear glands enlarged; gave hydrarg. bichloride 1-16 grain and potassium iodid. 2 grains three times a day; nothing else save bismuth subnitrate and minute doses of calomel for bowels.

Child soon began to regain health and strength; the diarrhœa ceased, and it made a rapid recovery.

In patients weak and anæmic, with failing strength, a natural hesitancy arises to giving so notorious tissue wasters as kalium iodide and mercury; but, let the syphilitic diathesis exist, they will usually produce so marked an improvement as to almost entitle them to the name of "tonic." Other physicians may differ with me as to the utility of these drugs, but, be their ideas regarding the proper treatment of syphilis what they may, I am confident that every practitioner will find it vastly to his own and his patients' interest for him to recognize and, whenever possible, treat the syphilitic diathesis.

Among all classes of children, and those of the colored race in particular, is to be found a large class of subjects that, while never developing rickets proper, are more or less victims of the same peculiar form of malnutrition. While this diathesis is, as a rule, comparatively easily eradicated, it exerts a very pernicious effect on the diseases of childhood. Bowel complaints, continued fevers, pulmonary diseases, and so forth, all present a much less favorable prognosis when associated with it. These little cases present enlarged joints, the cranial bones are thickened at the sutures, the bones of the fore-arm appear especially enlarged at the wrist joint; the limbs, as a rule, are thin and ill-formed. Where the condition verges upon rachitis proper, the sternum is often prominent, the pigeon-breast appearance. These patients are in a state of lowered vitality that renders them unfit to resist the onset of acute disease. Blood and tissue assimilate but poorly, have done so for a long time and are necessarily weak and enfeebled. When any intercurrent disease attacks such an organism it either rapidly succumbs, or, if the first onset be successfully resisted, nature seems unable to do more than maintain a feeble and unequal struggle, sometimes successful, as often failing; if the former, the result is apt to be tardy and long delayed. To treat such cases successfully, the assimilative and nutritive powers of the patient must be improved. The greatest care must be paid to the diet and digestive organs. Iron, lead, liver oil, phosphorous and the other remedies, as well as the hygienic measures suitable for uncomplicated rachitis, are, when available, all useful. I am in the habit of prescribing McArthur's syrup hypophos. comp., not that I doubt the equal efficacy of other preparations, but because I have found none more prompt or more efficacious than this. Of all drugs, considering its reputation at the South, I have been least satisfied with the iodide of iron. To illustrate the value of treating this diathesis during some intercurrent attacks, I will give you the following case: A. C., colored, aged two and one-half years. Saw case February 14th, 1890. Found child with well marked broncho-pneumonia of upper lobe of right lung. Bowels diarrhœaic and stomach excessively irritable. Well marked rachitic diathesis. Neglected the diathesis, and put on treatment for pneumonia alone. Case lingered without improvement until February 21st, when I stopped all treatment directed to the lung except the use of the muriate of ammonia, and began the use of McArthur's syrp. hypophos. Improvement was soon observed and recovery was rapid and complete. Continued hypophosphites, and now the child enjoys better health than ever before in its life.

The strumous diathesis resembles the rachitic in that its victim's tissues are ill-nourished and function but poorly. It always means a weak, debilitated and nervous system, which, when attacked by serious disease, can present but comparatively little hope of ultimate recovery. The condition can often be eradicated, or at least alleviated, but the change is slow, and time, which is everything, is too often denied. Two separate types of this diathesis may be recognized—the one usually occurring in those whose ancestors have lived in ease, if not in luxury;' 'the pretty struma;" the skin is pale, waxy and transparent; thorax badly formed; usually precocious and early and badly matured; a nervous and irritable disposition; easily falling before acute disease. The other is the child of poverty; the result of long continued bad hygienic surroundings; thick, muddy skin; thick lips; enlarged glands; often enlarged abdomens; minds usually dull and sluggish. In these cases the intercellular spaces are blocked by numerous large lymph-cells, and absorption takes place but poorly. Inflammations tend to become chronic and are especially liable to suppuration. Nutritive and reproductive changes are sluggish and ill performed. It may with truth be said that the strumous diathesis greatly adds to the death rate of every known disease. When called to treat any disease, no matter what, in a person of this diathesis, an effort should always be made to eradicate or alleviate it in so far as may be possible. In a vast per cent. of diseases no time is allowed in which to successfully accomplish this; but the effort can do no harm. Put the patient in the best hygienic surroundings possible. Above all, remove him as far as possible from soil dampness and impure air. Cod liver oil, iron and similar medicinal agents are to be given whenever practicable. Diet should be simple but of the most nutritious kind, and of the greatest importance is keeping the digestive organs in the best possible condition. Alcohol acts well in this diathesis, but should never be abused. To recognize the condition is usually easy, and the appearance of the patient and the family history will usually point the physician to a proper conclusion. If he should be in doubt, he may remember that no harm is ever done by improving the hygienic surroundings and nutrition of a patient.

The physician in general practice sees the gouty diathesis more often than he is aware. Those who are its victims are usually of strong frames, ruddy cheeks, large limbs and bones, hair gray early, though the whole appearance indicating health. The history of family and mode of life are valuable in discovering the condition. Heredity has been estimated in from forty to ninety per cent., and, as a rule, these patients are high livers and more or less addicted to alcoholic drinks. Such an apparent state of health is deceptive and but masks a liability to the gravest of organic diseases. Such a patient will come to his physician complaining of indigestion, a slight constant headache, continual irritability and similar little symptoms, and too often he gets nothing better than some pepsin or a "liver pill." It is exactly in this state that the recognition of the diathesis is most valuable from a practical standpoint. The liver is inactive, the blood loaded with uric acid, and if not removed, what produced a headache may produce a cinhotic kidney. If the patient is, as is most often the case, of sedentary habits, he should take exercise. He should be restricted to a simple and carefully selected diet, and an embargo put upon his alcohol. To stimulate the stomach digestion and the liver I am in the habit of using nitro-muriatic acid in from three to six minim doses after each meal. Even where organic disease has been induced by bearing in mind the diathesis and regulating diet and mode of life, accordingly a factor of vital importance in the treatment of the affection is secured. This advice in a case where the diathesis is often the obvious cause of the disease may seem unnecessary, but I am convinced that, even where the presence of the diathesis is fully recognized, these important measures are too often neglected

A powerful, yet insidious enemy, is malaria. Varied are its disguises, but its purpose for evil is always the same. It often lies in ambush during months and even years of apparent health, and then when other disease has attacked the organism, either springs openly from its covert or, still in partial ambuscade, wounds and disables with noiseless dart. There is scarcely a known symptom of which its victims may not complain. Yet, while always possessing a depressing and vitiating influence upon the nervous and other vital forces, it more or less impresses its peculiarities upon every disease that attacks a system in which it has previously found lodgement. Its victims usually present a digestion disturbed, bad taste, the skin yellow, or sallow, periodicity, and a spleen more or less enlarged, and tender are its trade-marks. No matter what intercurrent disease is present, a close attention to the affection will show exacerbations of certain symptoms, febrile or otherwise, coming on at stated intervals. Very often this periodicity exhibits itself in some kind of regularly recurring nervous phenomena. Add to this an enlarged spleen, rarely unrecognized by careful examination, and the history or suspicion of a more or less prolonged exposure to malarial influences, and the existence of this cachexia is scarcely to be doubted. In such cases quinine, wherever tolerance is possible, should form the basis of the treatment, no matter what the disease in hand.

But a short time since, I paid a hurried country call to a child some ten years of age; found it suffering with a dysenteric diarrhœa; prescribed for such. A week later the father came to me saying the child was no better; careful questioning elicited the fact that the disease showed a tendency to become much more severe on alternate days. Ordered quinine ; fifteen grains a day. A week later the father reported the child convalescent. Arsenic and Iron will often form important adjuncts in the treatment.

In chronic affections, influenced by the malarial cachexia, I have found a pill, composed of two-thirds of a grain each of pulv. sanguinariae, pulv. capsici and quinine sulphate given three times a day, to be of the greatest benefit. In districts where malaria is extremely prevalent it is the custom, and rightly so, to give quinine for almost everything. In those localities where the cachexia is infrequent or even rare, it behooves the physician no less to recognize and treat it, for it is a condition that, while usually easily placated by attention, most imperatively demands to be noticed.

It might almost be said that scurvy is a thing of the past. But the scorbutic cachexia is a thing of by no means uncommon occurrence. It especially occurs among those classes who eat but few fresh vegetables. After bad seasons, seasons of much rain or long drouth, or after a long winter it is not uncommon to find it among our lower classes who are unable or unwilling to procure vegetables. Persons in this state are not only more liable to disease, especially bowel complaints, but they are less able to contend with them than when in their normal states. Such individuals are usually rather emaciated, skin putty-colored, knees perhaps slightly swollen, lips pale and in striking contrast with the gums which are red, tender, more or less swollen and easily bleed. A diagnosis of these cases by treatment is usually easy. Give them lemons, say one three times a day. If a scorbutic taint be the underlying cause of the disease a rapid amelioration of symptoms will usually occur. If lemons be unavailable, onions will form a treatment more pleasant in result than in odor. The scorbutic cachexia is more common in higher latitudes than in our own, but it occurs here with sufficient frequency to make it desirable for the physician to remember the possibility of its occurrence; it will be useful to him occasionally in cases that threaten to be obstinate.

Some of the Diathesis and cachexiae never occur together, as the strumous and gouty. More often they are liable to exist in the same person; especially the syphilitic with the rest. Where this occurs it must rest with the judgment and ingenuity of the physician, as to the proper treatment. It is not the wish of the author to imply that the recognition of a Diathesis during the course of an intercurrent disease indicates that the treatment of the Diathesis should supercede that of the disease. As a rule, it should be merely an addition. In those cases where the Diathesis is more or less directly responsible for the attack in question, its treatment may, to a greater or less extent, be relied upon for the cure; the judgment of the attending physician must always decide to what degree it should supercede other treatment. But let me reiterate the Diathesis should receive *some* treatment except in those cases where the rapidity and virulence of the acute attack precludes any hope of benefit from such measures. It is now universally admitted that few drugs are, in any sense, specific. Nature performs the cure; we can but strengthen and assist. With this fact before us, who can doubt that for her to most successfully complete a cure she should be in the smallest possible degree handicapped. Therefore it behooves us to eradicate in so far as may be possible all vitiating influences. All Diatheses are abnormal; they weaken nature and render her unfit for her proper functions. And the man who recognizes such a condition and does not seek to remove it merely because he has some other more acute, and therefore more vociferous disease before him, practically says to nature: "You are, while weak, debilitated, unfit I admit, for severe trials, compelled to struggle against this powerful enemy. Well, overcome this enemy, and then perhaps I will aid you to regain your normal strength." And should nature prove unequal to the task and fall by the wayside, this man will piously raise his hands and say, "It is the will of God." If he should fail to recognize the Diathesis it redounds to the glory of his ignorance or of his carelessness, not to the brightness of his medical reputation. Physicians may differ as to the best methods to be pursued in the treatment of these conditions, but I think that none will deny the utility of removing in so far as is possible their influence upon the system, especially where that system is acutely diseased. Idiosyncrasy and temperament might often be written, "Diathesis."

Superior success in this "old family physician," is often due to the conscious or perhaps as often unconscious recognition of some Diathesis that exists in the family, which he invariably treats and is apt to call the families' peculiarity. And finally, it is my firm conviction that he who is mindful of the substratum of Diathesis, will be most successful in removing the super-structure of disease.

